# The Application of Projection Word Embeddings on Medical Records Scoring System

**DOI:** 10.3390/healthcare9101298

**Published:** 2021-09-29

**Authors:** Chin Lin, Yung-Tsai Lee, Feng-Jen Wu, Shing-An Lin, Chia-Jung Hsu, Chia-Cheng Lee, Dung-Jang Tsai, Wen-Hui Fang

**Affiliations:** 1 School of Medicine, National Defense Medical Center, Taipei 114, Taiwan; xup6fup0629@gmail.com; 2 School of Public Health, National Defense Medical Center, Taipei 114, Taiwan; 3 Graduate Institute of Life Sciences, National Defense Medical Center, Taipei 114, Taiwan; 4 Artificial Intelligence of Things Center, Tri-Service General Hospital, National Defense Medical Center, Taipei 114, Taiwan; 5 Division of Cardiovascular Surgery, Cheng Hsin Rehabilitation and Medical Center, Taipei 112, Taiwan; andrewytlee.cvs@gmail.com; 6 Department of Informatics, Taoyuan Armed Forces General Hospital, Taoyuan 325, Taiwan; army.afth@gmail.com; 7 Department of Medical Informatics, Tri-Service General Hospital, National Defense Medical Center, Taipei 114, Taiwan; beeverything@hotmail.com (S.-A.L.); jayronhh@gmail.com (C.-J.H.); lcgnet@gmail.com (C.-C.L.); 8 Division of Colorectal Surgery, Department of Surgery, Tri-Service General Hospital, National Defense Medical Center, Taipei 114, Taiwan; 9 Department of Family and Community Medicine, Department of Internal Medicine, Tri-Service General Hospital, National Defense Medical Center, Taipei 114, Taiwan

**Keywords:** medical records scoring, projection word embedding, long short-term memory, bidirectional encoder representations from transformers, artificial intelligence, natural language processing, electronic health records

## Abstract

Medical records scoring is important in a health care system. Artificial intelligence (AI) with projection word embeddings has been validated in its performance disease coding tasks, which maintain the vocabulary diversity of open internet databases and the medical terminology understanding of electronic health records (EHRs). We considered that an AI-enhanced system might be also applied to automatically score medical records. This study aimed to develop a series of deep learning models (DLMs) and validated their performance in medical records scoring task. We also analyzed the practical value of the best model. We used the admission medical records from the Tri-Services General Hospital during January 2016 to May 2020, which were scored by our visiting staffs with different levels from different departments. The medical records were scored ranged 0 to 10. All samples were divided into a training set (*n* = 74,959) and testing set (*n* = 152,730) based on time, which were used to train and validate the DLMs, respectively. The mean absolute error (MAE) was used to evaluate each DLM performance. In original AI medical record scoring, the predicted score by BERT architecture is closer to the actual reviewer score than the projection word embedding and LSTM architecture. The original MAE is 0.84 ± 0.27 using the BERT model, and the MAE is 1.00 ± 0.32 using the LSTM model. Linear mixed model can be used to improve the model performance, and the adjusted predicted score was closer compared to the original score. However, the project word embedding with the LSTM model (0.66 ± 0.39) provided better performance compared to BERT (0.70 ± 0.33) after linear mixed model enhancement (*p* < 0.001). In addition to comparing different architectures to score the medical records, this study further uses a mixed linear model to successfully adjust the AI medical record score to make it closer to the actual physician’s score.

## 1. Introduction

With the increasing advancement of technology, the data amount generated by humans is growing explosively [1]. Effectively taking advantage of these growing data may bring valuable information, which many successful cases from different industries [2] have already proved. However, the majority of these data are not structured [3], which cannot be directly used by traditional analytical methods. At the same time, it is expected to employ new algorithms to use these data to allow for stronger decision-making capacity [4,5]. In recent years, with the breakthrough developments of the deep neural network in diverse fields, we are already capable of directly analyzing data in the forms of videos, texts, and voices. Hence, the focus of researches is now to develop applications to solve practical problems.

The medical system is an important field that is very suitable to develop the abovementioned applications. Medical knowledge is accumulating quickly, making it more and more possible for doctors to have knowledge gaps [6], which may cause misdiagnoses and, thus, urgently need to be solved [7]. Computer-aided diagnosis systems have been greatly developed in recent years, aiming to solve this problem, yet unsuccessfully so far [8]. This is probably because the majority of medical data are non-structural data [9]; take cancer, for example, where about 96% of cancer diagnoses are made from pathological section reports, the data of which, however, are recorded in text descriptions and videos [10]. Thus, it is difficult for traditional models to link these original non-structural data with diagnosis information directly. With the advancement of artificial intelligence (AI) technology, the new generation of computer-aided diagnosis systems is expected to make great contributions to the intellectualization of medical systems. It can further eliminate human errors to increase the quality of medical care [11]. In 2012, AlexNet was the ILSVRC champion, leading the 3rd AI revolution [12]. Since then, more powerful deep learning models have been developed, such as VGGNet [13], Inception Net [14], ResNet [15], DenseNet [16], etc. This revolution led by deep learning has made enormous progress in image recognition tasks, driving breakthroughs in related research. Computer-aided diagnosis tools built based on deep learning technology have led to an increase in medical care quality [11]. Examples include lymph node metastasis detection [17], diabetic retinopathy detection [18], skin cancer classification [19], pneumonia detection [20], bleeding identification [21], etc. There have been over 300 studies (mostly in the last 2 years) using such technologies in medical image analysis [22]. It is worth mentioning that the most impressive capacity of deep learning technology is automatic feature extraction. With the precondition of a large database for annotation, it has been proven to reach, or even surpass, the level of human experts [15,23,24].

The current method to use a large amount of information from medical records is to code through recognition by experts and according to ICD (The International Statistical Classification of Diseases and Related Health Problems). This work is not only necessary for our national health insurance declaration system but may also be used in disease monitoring, hospital management, clinical studies, and policy planning. However, artificial classification is not only expensive but is also time-inefficient, which is the most important. For example, in disease monitoring, since the outbreak of infectious disease will cause large casualties [25], many countries have developed their disease monitoring systems specifically aiming at contagious diseases, such as the Real-time Outbreak and Disease Surveillance (RODS) system [26]. To ensure time efficiency, this system stipulates emergency physicians to input data within required time limits when identifying notifiable diseases, making it hard to be promoted to other diseases. With the advancement of data science, it has been universally expected that an automatic disease interpretation model can be developed to solve the high-cost and time-inefficient problems of artificial interpretation.

Due to the popularization of medical records electronization, a great number of studies have attempted to use this information for text mining and ICD code classification. The current technology primarily uses a bag-of-words model to standardize text medical records, then uses a support vector machine (SVM), random forest tree, and other classifiers for diagnosis classification [27,28,29,30,31]. However, previous studies have found that these methods were incapable of accurate diagnosis classification because of the particularity and diversity of clinical terms, where synonyms need to be properly processed before data preprocessing [10]. A complete medical dictionary integrates the currently recommended forms of clinical terms; yet, it is almost impossible due to the complexity of clinical terms. Therefore, traditional automatic classification programs can hardly make significant progress. In addition, the bag-of-words model treats different characters as different features and counts the number of features in one article. Although this makes it possible to use a dictionary to handle the synonym problem, similar characters would be considered two different features. Thus, the number of features integrated by the bag-of-words model will be strikingly huge, causing a curse of dimensionality when classified by subsequent classifiers, leading to inefficiency and slow progress of traditional algorithms.

Other than classification efficiency, the greatest challenge for traditional algorithms is new diseases. For instance, there was an H1N1 outbreak in 2009, with related cases that had never been recorded before 2008. Traditional classification algorithms are completely unable to perform proper classification of newly emerged words [27,28,29,30,31]. This disadvantage makes it absolutely impossible for traditional methods to reach full automation. Regarding this issue, we proposed word embedding as a technical breakthrough in disease classification. Since the 20th century, word embedding has been an important technology to allow computers to understand the semantic meaning further. Its core logic is hoping to characterize every single word into a vector in high-dimensional space and expecting similar vectors for similar characters/words to express semantic meaning [32,33]. The word2vec published by the Google team in 2013 is considered the most important breakthrough in recent word embedding studies. It has been verified to allow similar characters to have very high cosine similarity and very close Euclidean distance in vector space [34]. However, this technology has a disadvantage that, once applied, it converts an article into an unequal matrix, making it inapplicable for traditional classifiers, such as SVM and random forest trees. A general solution is to average or weighted average the word vector of all characters in an article as semanteme [35]. However, from the MultiGenre NLI (MultiNLI) Corpus competition release by the natural language research team of Stanford (https://nlp.stanford.edu/projects/snli/), we can still see that combining modern AI technology gives better efficiency to models. Language processing conducts analysis mostly based on Recurrent Neural Network (RNN) or Convolutional Neural Network (CNN). Its core principle is to use convolutional layer (does not have memory but can gradually integrate surrounding single-character information in higher-order features, requires more layers) or Long Short-Term Memory Unit (has short- and long-term memory, thus needing fewer layers) for feature extraction and is able to process information in matrix form [36]. CNN has become the primary method in all computer vision competitions. Its reason for success is a fuzzy matching technique of convolutional layer, allowing for integrating similar image features. We will be able to change the convolutional layer from recognizing similar image features to recognizing similar vocabularies through certain designs. Hence, CNN has been applied in text mining, such as semantic classification [37], short sentence searching [38], and chapter analysis [39], and has shown considerably good efficiency. In the most recent study, Bidirectional Encoder Representations from Transformers (BERT), developed by Google, has swept all kinds of natural language process competitions [40]. Yet, its core is still good work/sentence/paragraph embedding. Generally speaking, combining good embedding technology with modern deep learning neural networks is undoubtedly the best option for current natural language processing tasks.

Our team has already applied it in disease classification of discharge record summaries and proved that it compared with traditional models. AI model with combined word embedding model and CNN reduces 30% error rate in disease classification tasks, makes modeling easier by avoiding troublesome text integration preprocessing, and learns external language resources through unmonitored learning to integrate similarity among clinical clauses [41]. However, although the combination of word embedding and CNN is better in disease classification tasks than traditional methods, its accuracy still cannot be compared with humans. One of the reasons is the error in understanding the semantic meaning. Therefore, improving the word embedding model’s understanding of the meaning of medical terms might increase its subsequent analytical efficiency [42]. There are two studies that have evaluated the application of word embedding models trained by different resources on biomedical NLP and found EHR-trained word embedding could better capture semantic property [43,44]. On the other hand, external data resources have a neglected advantage in that the vocabulary diversity of external internet data resources is far more than that of internal task database. This advantage will greatly affect real disease coding tasks. Hence, an embedded training process needs to be developed to maintain the vocabulary diversity of internet resources and medical terms’ understanding of the internal task database. A recent word embedding comparison study showed that EHR-trained work embedding could usually better capture medical semantic meaning [43]. Even the research team of abroad Mayo Clinic uses an EHR with a large amount of data. The total number of words is only about 100,000, the vocabulary diversity of which is still far less than the external database [43,44]. This is due to the lack of some rare diseases and periodic diseases, such as the 2003 SARS outbreak and the 2009 H1N1 outbreak. Therefore, EHR-trained word embedding models are unable to include enough vocabulary. For this reason, our team developed a projection word embedding model that has the vocabulary diversity of Wikipedia/PubMed, as well as an understanding of medical terms in EHR [45].

A medical record is a historical record and also the foundation of a patient’s medical care. It records the patient’s conditions, reasons, results of examinations/tests, treatment methods, and results during care processes. It integrates and analyzes patients’ related information, presents the executive ground of medical decisions, and even affects national health policy. The basic purpose of medical records is to remind oneself or other medical care colleagues of a patient’s daily conditions and attending physician’s current thoughts. When medical treatment is being performed, the medical record serves as the communication tool among physicians and means for continuous treatment. In other words, the medical record is the only text material that records a patient’s conditions and focuses on all medical care personnel. A medical record is an index of medical care quality reflecting a physician’s clinical thinking and diagnostic basis. It serves as the reference for learning, research, and education. Meanwhile, it also serves as the evidence for medical disputes to clarify the attribution of liabilities. The medical record is the foundation of patient care as it records the contents of patient care provided by medical personnel. Thus, all results obtained from observation or examination can be found on the medical record. Therefore, any change in the patient’s condition can be found from the medical record so that the patient’s current condition can be evaluated for suitable treatments. Moreover, communication with a patient should also be included in the medical record so that medical personnel can learn the patient’s expectations on the treatment, resulting in a closer doctor-patient relationship. For other professionals, a detailed medical record saves a lot of communication time and avoids misunderstanding or missing the patient’s previous conditions that may lead to mistreatment.

The content of medical records also has legal effects. It is the basis of insurance benefits and even affects national health policy. For example, public health studies usually need to include case information under national health insurance, and, through studying a large number of medical records, such studies can help public health researchers and medical officials to establish more suitable public health decisions and administrative rules that protect the rights and interests of both doctors and patients. Clinical decision-making guides formulated by many specialized medical associations also used information from medical records. The implicit demographic information from these medical records is also collected at the national level and published as national health demographic information to compare with other countries so as to serve as a way to communicate and learn from each other for mutual benefits.

In this study, as shown in the graphical abstract, a scoring database was established by experts performing scoring on medical records. An AI model was trained to learn experts’ scoring logics so as to screen high-quality medical record summaries. In contrast, the database made up of which will have the chance to promote the establishment of other subsequent AI models, improve model accuracy, and serve as a teaching example to improve medical education efficiency.

## 2. Method

### 2.1. Data Source

In this study, inpatient medical records from Tri-Service General Hospital from 1 January 2016 to 31 December 2019 were used as the basic database, which was ethically approved by institutional review board (IRB NO. A202005104). Physicians of different levels from different departments were invited for medical records summary scoring. Scoring dimensions include different indexes, based on clinical writing standards, it contains 12 scoring items from each detailed structure of the QNOTE scale’s inpatient record, including chief of complaint, history of the present illness, problem list, past medical history, medications, adverse drug reactions and allergies, social and family history, review of systems, physical findings, assessment, plan of care, and follow-up information. The completeness of each item’s record, as well as the 5 structures (completeness, correctness, concordance, plausibility, and currency) of electronic medical records’ examination information, are evaluated in 5 levels of the Likert scale: strongly disagree, disagree, no comment (not agree nor disagree), agree, and strongly agree. Specialists from different departments were required to review 227,689 medical records and preliminarily score them on a 10-point Likert scale based on the average of above 5 structures. These scores were then used as the training target of the AI model to represent medical record writing quality. All samples were divided into a training set (*n* = 74,959) and testing set (*n* = 152,730) based on time, and then they were evaluated by different departments. Data of the testing set was compared with the actual scores for analysis, and MAE from the Likert scale was used as the evaluation index for model performance. In the end, the aforementioned model was applied in Tri-Service General Hospital. A medical record auto-scoring system was established in the hospital so as to screen high-quality medical records for future teaching and research studies.

### 2.2. AI Algorithm

The collected medical records and various writing quality indicators can be used for artificial intelligence model training. The model architecture uses the word embedding and LSTM model developed by our team. The word embedding also uses the projection word embedding comparison table to perform single-character conversion mathematical vectors and uses the entire input article as the input matrix. We used projection word embedding to construct a deep convolutional network model to enable the network to integrate the transformed semantic vectors and extract written medical records based on different word combinations. First, we used the word embedding comparison table trained by Wikipedia and PubMed library, and then we used EHR to perform projection word embedding training. Next, we connected the converted text matrix in parallel so that the network can refer to two different word embedding sources simultaneously. In addition, we used different word embeddings separately as conversion sources to compare their effects on prediction performance. 

#### 2.2.1. Long Short-Term Memory (LSTM)

In RNN, the output can be given back to the network as input, thereby creating a loop structure. RNNs are trained through backpropagation. In the process of backpropagation, RNN will encounter the problem of vanishing gradient. We use the gradient to update the weight of the neural network. The problem of vanishing gradient is when the gradient shrinks as it propagates backwards in time. Therefore, the layers that obtain small gradients will not learn but will, instead, cause the network to have short-term memory.

The LSTM architecture was introduced by Hochreiter and Schmidhuber [46] to alleviate the problem of vanishing gradients. LSTMs can use a mechanism called gates to learn long-term dependencies. These gates can learn which information in the sequence is important to keep or discard. LSTMs have three gates: input, forget, and output. This is the core of the LSTM model, where pointwise addition and multiplication are performed to add or delete information from the memory. These operations are performed using the input and forget gate of the LSTM block, which also contains the output “tanh” activation function. In addition to using the original architecture and model parameters, the other settings are Epochs = 20, Batch size = 300, and Learning rate = 0.001.

#### 2.2.2. Bidirectional Encoder Representation from Transformers (BERT)

Other than the original word embedding and LSTM architecture, BERT architecture was also used for feature extraction. BERT is a recent attention-based model with a bidirectional Transformer network that was pre-trained on a large corpus. This pre-trained model is then effectively used to solve various language tasks with fine-tuning [40,47]. In brief terms, the task-specific BERT architecture represents input text as sequential tokens. The input representation is generated with the sum of the token embeddings, the segmentation embeddings and the position embeddings [40]. For a classification task, the first word in the sequence is a unique token which is denoted with [CLS]. An encoder layer is followed with a fully-connected layer at the [CLS] position. Finally, a softmax layer is used as the aggregator for classification purposes [47]. If the NLP task has pair of sentences as in question-answer case, the sentence pairs may be separated with another special token [SEP]. BERT multilingual base model (cased) is used as transfer feature learning, and other parameters are set to Epochs = 30, Batch size = 32, and Learning rate = 0.00001.

Through these two methods, we can enable the network to learn the semantic meanings of different individual characters. We can also let the network learn from different texts, such as from Wikipedia and PubMed. Then, through EHR for Fine-tune retraining, the BERT architecture that has finished learning only needs to change from predicting its context output to predicting the categories of multiple medical record quality dimensions; then, it can be trained with medical record information. 

### 2.3. Linear Mixed Model Function for Medical Records Scoring Prediction

Suppose data are collected from m independent groups of observations (called clusters or subjects in longitudinal data).

(1) $$Y_{m}=X_{m}B_{m}+e_{m}.$$

Here, *Y_m_* is an *n* × 1 vector of the dependent variable for patient *m*, and *X_i_* is an *n* × *q* matrix of all the independent variables for patient *m*. *B_m_* is a *q* × 1 unknown vector of regression coefficients, and *e_m_* is an *n* × 1 vector of residuals. This results in a multi-level mixed model with random effects for all samples, which is expressed as

(2) Y=XB+Zu+e,

where *Z* is a matrix of known constants included in the information of the independent variables with random effects, and *u* is a matrix of random effects for all patients.

The best linear unbiased prediction (BLUP) is important for predicting the medical record score in each patient, and it can be calculated by following the steps in [48].

*Y_m_* is an *n* × 1 vector of the dependent variable for patient *m*, and *X_i_* is an *n* × *q* matrix of all independent variables for patient *m*. Moreover, *Z_m_* is an *n* × *p* matrix of independent variables with random effects for patient *m*. These matrices contain the observed data and are defined as

(3) $$Y_{m}=\left[\begin{array}{c} y_{1,m}\\ y_{2,m}\\ \ldots \\ y_{n,m} \end{array}\right],\;X_{m}=\left[\begin{array}{cccc} 1 & x_{1,1,m} & \ldots & x_{1,q-1,m}\\ 1 & x_{2,1,m} & \ldots & x_{2,q-1,m}\\ \ldots & \ldots & \ldots & \ldots \\ x_{1} & x_{n,1,m} & \ldots & x_{n,q-1,m} \end{array}\right],\;Z_{m}=\left[\begin{array}{cccc} 1 & x_{1,1,m} & \ldots & x_{1,p-1,m}\\ 1 & x_{2,1,m} & \ldots & x_{2,p-1,m}\\ \ldots & \ldots & \ldots & \ldots \\ x_{1} & x_{n,1,m} & \ldots & x_{n,p-1,m} \end{array}\right].$$

After building the prediction tool, we have the *G* matrix, *B* vector and *σ*^2^. *G* is a variance co-variance matrix of the random effects (*p* × *p*), and *B* is the fixed effect coefficients vector (*q* × 1). *σ*^2^ is the variance of the residuals. We can calculate a matrix *R* (*n* × *n*) using

(4) $$G=\left[\begin{array}{cccc} \tau_{1}^{2} & \tau_{12} & \ldots & \tau_{1p}\\ \tau_{12} & \tau_{2}^{2} & \ldots & \tau_{2p}\\ \ldots & \ldots & \ldots & \ldots \\ \tau_{1p} & \tau_{2p} & \ldots & \tau_{p}^{2} \end{array}\right],\;B=\left[\begin{array}{c} b_{0}\\ b_{1}\\ \ldots \\ b_{q-1} \end{array}\right],\;R=\sigma^{2}I_{n\times n}=\left[\begin{array}{cccc} \sigma^{2} & 0 & \ldots & 0\\ 0 & \sigma^{2} & \ldots & 0\\ \ldots & \ldots & \ldots & \ldots \\ 0 & 0 & \ldots & \sigma^{2} \end{array}\right].$$

If the independence assumption holds (i.e., $\left[\begin{array}{c} u\\ e \end{array}\right]\sim N\left(\left[\begin{array}{c} 0\\ 0 \end{array}\right],\left[\begin{array}{cc} G & 0\\ 0 & R \end{array}\right]\right)$), then we can calculate the variance co-variance matrix (Σ*_m_*) of *Y_m_* using

(5) $$\Sigma_{m}=Z_{m}GZ_{m}^{T}+R.$$

Finally, the BLUP of the random effect in patient *m* can be estimated using

(6) $$BLUP_{m}=GZ_{m}^{T}\Sigma_{m}^{-1}\left(Y_{m}-X_{m}B\right).$$

We can estimate the regression coefficients (*B_m_*) in patient *m* based on the above result, and *B_m_* can be used to predict the disease progression. *B_m_* can be calculated using

(7) $$B_{m}=B+BLUP_{m}$$

Note that this calculation cannot make direct forecasts without the co-variable values. Thus, the co-variables information at the time of interest must be generated. We propose two methods for generating this information: (1) assume consistency between the last time and the time of interest and (2) predict the linear expectations. We will assess these methods in our analysis. Unquestionably, clinicians can use the most reasonable values based on their judgment to predict the co-variables at the time of interest. In summary, we can combine this method with population information to predict the medical record score.

### 2.4. Evaluation Criteria

We evaluated the generalization performance of each model in the training and testing samples. Mean absolute error (*MAE*) were used to compare the performance of the models, as follows:

(8) $$MAE=\frac{\sum_{i=1}^{N}\left|y_{i}-{\hat{y}}_{i}\right|}{N}\;.$$

## 3. Results

**Figure 1:**
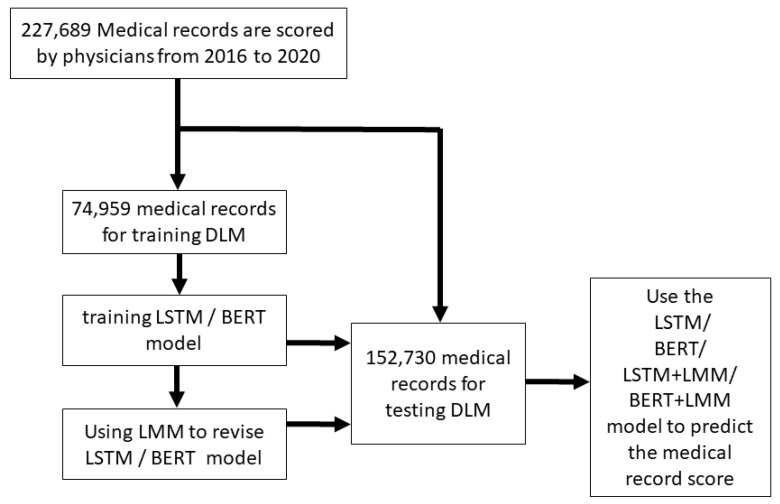
Training and testing sets generation. Schematic of the data set creation and analysis strategy, which was devised to assure a robust and reliable data set for training and testing of the network. Once a medical records data were placed in one of the data sets, that individual’s data were used only in that set, avoiding ‘cross-contamination’ among the training and testing sets. The details of the flow chart and how each of the data sets was used are described in the Methods.

The research scheme is shown in Figure 1, where a total of 227,689 medical records were scored by experts. In AI model training, the medical records were divided into the training set and testing set based on year, where 74,959 records were used to establish BERT and LSTM models, and 152,730 records were used to test record scoring. LMM was then employed to modify BERT and LSTM to establish another two models. In the end, MAE was used to compare the four models’ efficiencies in predicting medical record scores.

Table 1 shows the distribution of medical records in different departments. It can be seen that 74,959 records were included for modeling, and then 152,730 records were used for prediction. The average score from experts was 7.24 ± 1.02 for the training set and 7.67 ± 0.84 for the testing set; after BERT and LSTM modeling of medical record scoring, the average score of BERT prediction in the testing set was 7.47 ± 0.89, and 7.15 ± 1.05 for LSTM. After training through the BERT and LSTM models, the artificial intelligence model had already scored the medical records.

Our team’s projection word embedding model allowed the model to have both the vocabulary diversity of Wikipedia/PubMed and an understanding of medical terms in EHR. The concept of projection word embedding used the results of our previous studies, a concept in linear algebra that projects through matrix multiplication to allow all coordinates to convert into a new coordinate system. Such conversion changes the correlation of certain points while at the same time maintaining all current coordinates. In addition to the original projection word embedding and LSTM architecture, we attempted to use BERT architecture for feature extraction. BERT stands for Bidirectional Encoder Representations from Transformers, the elementary unit of BERT architecture is the encoder’s Multi-Head Self-Attention Layer in the transformer. In contrast, the overall architecture of BERT is stacked by a bidirectional Transformer Encoder Layer. As shown in Table 2, in general, on the ground of experts’ scoring, the trained scoring model BERT had a prediction score of 7.49 ± 0.28. In contrast, LSTM had 7.17 ± 0.31; after modification by the linear mixed model (LMM), BERT’s and LSTM’s prediction scores were 7.36 ± 0.56 and 7.33 ± 0.65, respectively. After layering different departments, such as internal medicine, surgery, obstetrics, and pediatrics, it can be learned that BERT all had higher prediction scores than LSTM, while, after LMM modification, all LSTM prediction scores increased. Through further looking into different departments, it was found that most departments’ BERT prediction scores were higher than that of LSTM, and the latter increased after LMM modification.

**Figure 2:**
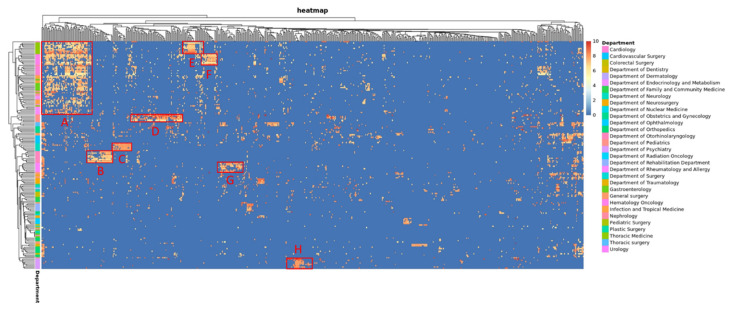
Heat map of medical record scores from scoring and scored physicians. *X*-axis: physicians who wrote the medical records; *Y*-axis: scoring physicians and their departments. A redder grid means record scoring physicians give a higher score to record writing physicians. There are clusters in some areas; thus, we put out some blocks and observe the block (A to H) characteristics in Table 4.

It can be learned from Table 3 that, when reviewer physicians’ scores and AI scores were calculated using mean absolute error (MAE), both BERT and LSTM AI scores were 0.6~1.3 points lower than reviewer physicians’ scores; thus, the linear mixed model (LMM) was introduced for modification, thereby reducing the score difference to 0.3~1 points, showing a significant reduction (*p* < 0.001) in score difference. The reason for the modification using LMM is that an ordinary linear regression contains only two influencing factors: fixed effect and noise. The latter is a random factor not considered in our model, while the former are those predictable factors that can also be completely divided. The AI scoring of medical records after modification by LMM is also more realistic. After department layering, it was found that, in some departments, LMM-modified MAE was not significantly reduced comparing with the original MAE. Hence, experts’ scores were made into a heat map (Figure 2), where it was found that some groups of scoring physicians and scored physicians had closer scores, and were separately analyzed. In Table 4, medical record prediction scores and MAE are analyzed from Block A to H, respectively, and, except for block F, most blocks had similar record scores with previous results, and the MAE of LSTM prediction scores significantly reduced (*p* < 0.05) after LMM modification.

**Figure 3:**
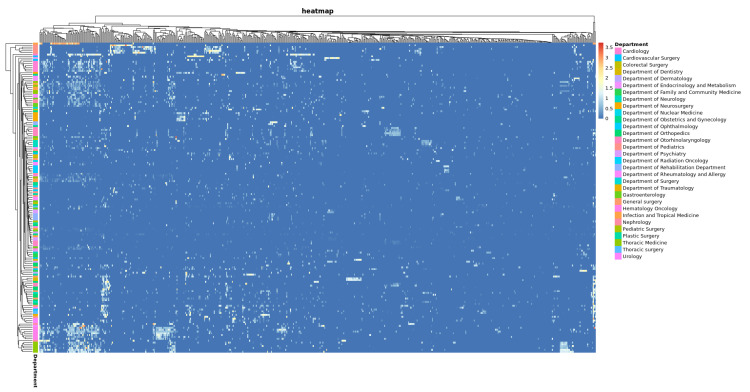
MAE heat map of LMM-modified LSTM prediction scores from scoring and scored physicians. *X*-axis: physicians who wrote the medical records; *Y*-axis: scoring physicians and their departments. By subtracting the MAE of the original score from the LMM modified LSTM prediction score, and using the MAE and coring physicians to conduct a heat map analysis, it can be found that some reviewer scores are on the high side.

**Figure 4:**
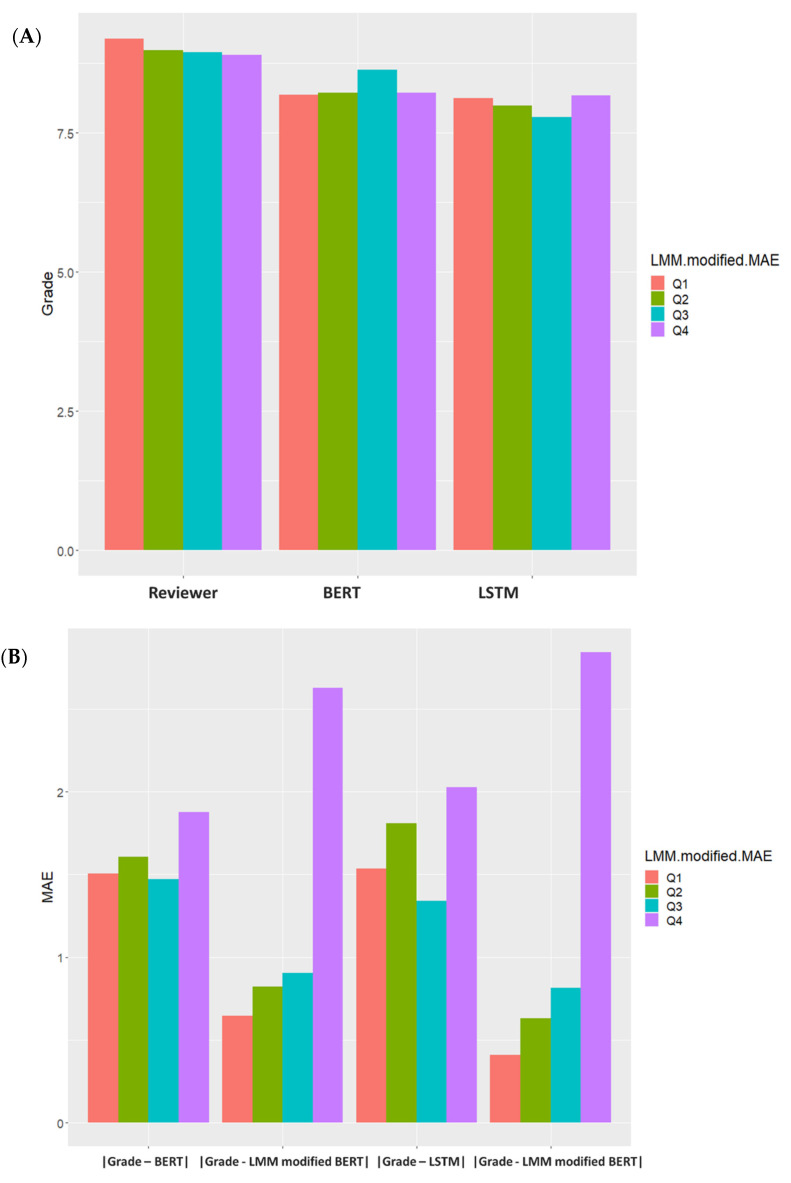
Using LMM modified MAE (Grade-LMM modified LSTM) for interquartile range grouping. (**A**): Compare the scores of Experts, BERT and LSTM. *Y*-axis: medical record scores, *X*-axis: Experts’ score, BERT prediction score, LSTM prediction score. (**B**): Compare the original MAE with the LMM modified MAE. *Y*-axis: mean absolute error (MAE), *X*-axis: |Grade—BERT|, |Grade—LMM modified BERT|, |Grade—LSTM|, |Grade—LMM modified BERT| for model efficiency evaluation. The LMM modified MAE (Grade-LMM modified LSTM) is grouped by interquartile range and divided into Q1, Q2, Q3, and Q4.

In spite of this, we were still unable to identify the reason why the MAE of certain departments had no significant reduction after LMM modification. Thus, heat map analysis was performed on LMM-modified LSTM prediction scores. Figure 3 shows that some reviewers’ LMM-modified LSTM prediction scores had relatively greater MAE. After grouping using LMM modified MAE (Grade-LMM modified LSTM), experts’ scores were close among groups, but BERT and LSTM prediction scores were lower than the original experts’ scores. In Figure 4, We further using MAE to evaluate model efficiency, and then comparing MAE (|Grade—LMM modified BERT|, |Grade—LMM modified BERT|) of LMM-modified BERT or LSTM with the MAE (|Grade—BERT|, |Grade—LSTM|) of the original BERT or LSTM, it was found MAE was effectively reduced through LMM modification in Q1~Q3, but not in Q4. Thus, it is suspected that some scoring physicians in Q4 may have scored incorrectly.

## 4. Discussion

In this study, the projection word embedding model was used to develop an AI system to evaluate the writing quality of inpatient medical records. The AI system is already capable of accurate classification to level 3 ICD-10 coding, combined with results from previous studies. Since level 3 coding is already at the disease level, subsequent coding will all just be remarks (such as location), and reaching such a level will allow for the possibility of full automation of common disease classification tasks, as well as extraction of disease features from other medical descriptions, through this algorithm. In addition to the original word embedding and LSTM architecture, BERT architecture was also employed to extract disease features for medical record scoring. LMM was further used for modification to get AI scores closer to actual reviewer physicians’ scores. Moreover, it was also identified that some physicians over-scored medical records. If these scoring standards can be improved in the future, a better medical writing quality could be expected.

In addition, why is the quality of medical record writing so important? Because the medical record is the historical record of the patient’s health care; it is also the basis of care, and its content records the patient’s condition during the care process, the reason and result of the inspection, and the treatment method and result. In recent studies, it is feasible to use electronic health records (EHR) to predict disease risk, such as atrial fibrillation (AF) [49], coronary heart disease in patients with hypertension [50], fall risk [51], multiple sclerosis disease [52], and cervical cancer [53]. Over the past two decades, the investigation of genetic variation underlying disease susceptibility has increased considerably. Most notably, genome-wide association studies (GWAS) have investigated tens of millions of single-nucleotide polymorphisms (SNPs) for associations with complex diseases. However, results from numerous GWAS have revealed that the majority of statistically significantly associated genetic variants have small effects [54] and may not be predictive of disease risks [55], and many diseases are associated with tens of thousands of genetic variants [56]. These findings have led to the resurgence of the polygenic risk score (PRS), an aggregate measure of many genetic variants weighted by their individual effects on a given phenotype. However, epidemiologic studies are expensive and complex to run, which raises the question of whether a PRS could be developed and applied in a clinical setting using genetic data that are more readily available. Recently, some scholars proposed new ideas for developing and implementing PRS predictions using biobank-linked EHR data [57].

For the medical records scoring system, this not only saves doctors the time for scoring medical records but also can get feedback immediately after the writing is completed to improve the quality of medical record writing. In the past research, clinicians spent 3.7 h per day, or 37% of their work day, on EHR [58]. There was a marked reduction in EHR time with both clinician and resident seniority. Despite this improvement, the total time spent on EHR remained exceedingly high amongst even the most experienced physicians [58]. The significance of an increasing shift towards EHR is a growing paradigm that cannot be understated, particularly in the current era of healthcare, and there is increasing scrutiny on documentation [59,60]. These increased demands can lead to EHR fatigue and physician burnout. In a survey of a general internal medicine group, 38% reported feeling burnt out, with 60% citing high documentation pressure and 50% describing too much EHR time at home [61]. Burnout has been linked to an increased risk of resident’s wellbeing [62]. 

There are still some limitations for electronic medical records. First, this scoring system can only be used in our hospital because the medical record system of different hospitals do not talk to each other. Second, entering data into an EHR requires a doctor to spend a lot of time doing so, leading to most physicians experiencing burnout symptoms due to EMR-related workloads. Third, cyber-attacks are a perennial concern for EHRs. It is, therefore, imperative that cybersecurity is continually enhanced. Fourth, timing discrepancies occur in EHRs, and they can lead to serious clinical consequences.

In summary, combining projection word embedding and LSTM with LMM can give better prediction scores. This system can be used to assist medical record scoring so that young physicians can get immediate writing feedback, so as to improve the quality of medical record writing in my country and let the public, Medical units, and insurance units can all get better help. In the future, it may be possible to actively introduce such technologies into hospitals to achieve personalized precision medicine.

## Figures and Tables

**Table 1:** Medical records distribution and scoring in the training set and testing set of different departments.

	Training Set (*n* = 74,959)	Testing Set (*n* = 152,730)	*p*-Value
Department			<0.001 *
General surgery	4843 (6.5%)	10,504 (6.9%)	
Pleural surgery	1932 (2.6%)	3472 (2.3%)	
Cardiovascular surgery	3904 (5.2%)	8319 (5.4%)	
Colorectal & rectal surgery	491 (0.7%)	3479 (2.3%)	
Urology surgery	1330 (1.8%)	3313 (2.2%)	
Pediatric Surgery	99 (0.1%)	85 (0.1%)	
Plastic surgery	1748 (2.3%)	4009 (2.6%)	
Pulmonary Medicine	10,268 (13.7%)	19,065 (12.5%)	
Cardiology	2723 (3.6%)	4765 (3.1%)	
Nephrology	2473 (3.3%)	3749 (2.5%)	
Blood Oncology	9257 (12.3%)	17,110 (11.2%)	
Endocrine and metabolic	839 (1.1%)	1477 (1.0%)	
Gastroenterology	3861 (5.2%)	7372 (4.8%)	
Rheumatism, immunology and allergy	1247 (1.7%)	2624 (1.7%)	
Trauma	756 (1.0%)	940 (0.6%)	
Infection and Tropical Medicine	3701 (4.9%)	8488 (5.6%)	
Psychiatric department	6531 (8.7%)	14,331 (9.4%)	
Neurological department	3159 (4.2%)	7374 (4.8%)	
Pediatric department	1138 (1.5%)	2474 (1.6%)	
Dental department	1223 (1.6%)	2483 (1.6%)	
Surgery department	607 (0.8%)	817 (0.5%)	
Dermatology department	5 (0.0%)	109 (0.1%)	
ENT department	2388 (3.2%)	3907 (2.6%)	
Radiology	40 (0.1%)	175 (0.1%)	
Emergency department	0 (0.0%)	300 (0.2%)	
Family and Community Medicine	188 (0.3%)	655 (0.4%)	
Nuclear Medicine Department	144 (0.2%)	153 (0.1%)	
Neurosurgery	3219 (4.3%)	6937 (4.5%)	
Orthopedic department	3482 (4.6%)	7876 (5.2%)	
Obstetrics and Gynecology	1766 (2.4%)	3222 (2.1%)	
Ophthalmology department	607 (0.8%)	903 (0.6%)	
Rehabilitation department	990 (1.3%)	2243 (1.5%)	
Experts’ scores	7.24 ± 1.02	7.67 ± 0.84	<0.001 *
BERT prediction score		7.47 ± 0.89	
LSTM prediction score		7.15 ± 1.05	

*: *p*-value < 0.05.

**Table 2:** BERT and LSTM original prediction scores and LMM-modified scores.

	Experts’ Scores	BERT Prediction Scores	LSTM Prediction Scores	LMM-Modified BERT Prediction Scores	LMM-Modified LSTM Prediction Scores
**Overall**	7.69 ± 0.64	7.49 ± 0.28	7.17 ± 0.31	7.36 ± 0.56	7.33 ± 0.65
Internal medicine	7.49 ± 0.66	7.37 ± 0.21	7.01 ± 0.20	7.14 ± 0.56	7.08 ± 0.65
Surgery	7.78 ± 0.55	7.49 ± 0.22	7.16 ± 0.17	7.54 ± 0.43	7.54 ± 0.51
Obstetrics and pediatrics	8.08 ± 0.69	7.68 ± 0.31	7.37 ± 0.31	7.70 ± 0.61	7.68 ± 0.79
Other departments	7.76 ± 0.60	7.57 ± 0.33	7.32 ± 0.40	7.39 ± 0.53	7.37 ± 0.61
**Department**					
General surgery	7.69 ± 0.74	7.48 ± 0.53	7.26 ± 0.28	7.45 ± 0.56	7.45 ± 0.57
Pleural surgery	7.87 ± 0.25	7.55 ± 0.35	7.22 ± 0.16	7.55 ± 0.43	7.64 ± 0.48
Cardiovascular surgery	7.73 ± 0.56	7.38 ± 0.37	7.01 ± 0.05	7.34 ± 0.17	7.35 ± 0.34
Colorectal & rectal surgery	7.92 ± 0.18	7.73 ± 0.37	7.22 ± 0.16	7.87 ± 0.35	7.97 ± 0.40
Urology surgery	7.76 ± 0.18	7.48 ± 0.29	7.14 ± 0.09	7.54 ± 0.25	7.48 ± 0.37
Pediatric Surgery	6.16 ± NA	6.86 ± 0.50	7.09 ± NA	6.86 ± NA	6.65 ± NA
Plastic surgery	7.98 ± 0.08	7.58 ± 0.32	7.20 ± 0.15	7.65 ± 0.23	7.65 ± 0.29
Pulmonary Medicine	7.58 ± 0.83	7.30 ± 0.57	6.98 ± 0.19	7.26 ± 0.58	7.22 ± 0.65
Cardiology	7.19 ± 0.97	7.02 ± 0.64	6.99 ± 0.08	6.83 ± 0.68	6.75 ± 0.73
Nephrology	8.13 ± 0.69	7.54 ± 0.55	7.12 ± 0.06	7.42 ± 0.47	7.39 ± 0.60
Blood Oncology	7.21 ± 0.55	6.89 ± 0.50	6.71 ± 0.16	6.77 ± 0.52	6.71 ± 0.74
Endocrine and metabolic	7.64 ± 0.26	7.38 ± 0.35	7.17 ± 0.04	7.35 ± 0.44	7.25 ± 0.55
Gastroenterology	7.19 ± 0.25	7.15 ± 0.26	6.96 ± 0.12	7.16 ± 0.30	7.09 ± 0.33
Rheumatism, immunology and allergy	7.79 ± 0.21	7.33 ± 0.32	6.98 ± 0.14	7.29 ± 0.17	7.19 ± 0.22
Trauma	7.84 ± 1.32	7.39 ± 0.57	7.18 ± 0.02	7.21 ± 0.35	7.14 ± 0.47
Infection and Tropical Medicine	7.33 ± 0.53	7.09 ± 0.57	6.98 ± 0.07	6.94 ± 0.74	6.89 ± 0.87
Psychiatric department	8.41 ± 0.48	8.08 ± 0.47	8.00 ± 0.16	7.94 ± 0.59	7.94 ± 0.67
Neurological department	7.89 ± 0.24	7.62 ± 0.23	7.39 ± 0.06	7.60 ± 0.18	7.63 ± 0.25
Pediatric department	7.91 ± 0.85	7.51 ± 0.66	7.14 ± 0.10	7.52 ± 0.66	7.48 ± 0.93
Dental department	7.95 ± 0.25	7.05 ± 0.52	6.53 ± 0.09	6.89 ± 0.04	6.76 ± 0.04
Surgery department	7.81 ± NA	7.40 ± 0.26	7.14 ± NA	7.33 ± NA	7.25 ± NA
Dermatology department	8.58 ± NA	7.67 ± 0.64	6.83 ± NA	7.73 ± NA	7.85 ± NA
ENT department	7.37 ± 0.49	7.36 ± 0.38	7.29 ± 0.15	7.32 ± 0.47	7.37 ± 0.54
Radiology	6.85 ± NA	6.70 ± 0.17	6.67 ± NA	6.51 ± NA	6.57 ± NA
Family and Community Medicine	7.37 ± 0.41	7.19 ± 0.61	7.29 ± 0.09	6.91 ± 0.80	6.90 ± 1.15
Nuclear Medicine Department	8.76 ± NA	8.01 ± 0.45	7.54 ± NA	7.83 ± NA	8.02 ± NA
Neurosurgery	7.95 ± 0.49	7.59 ± 0.56	7.12 ± 0.07	7.78 ± 0.63	7.78 ± 0.75
Orthopedic department	7.38 ± 0.40	7.21 ± 0.34	7.09 ± 0.09	7.14 ± 0.38	7.11 ± 0.44
Obstetrics and Gynecology	8.31 ± 0.34	7.96 ± 0.41	7.67 ± 0.23	7.95 ± 0.49	7.96 ± 0.51
Ophthalmology department	7.86 ± 0.19	7.65 ± 0.26	7.56 ± 0.06	7.54 ± 0.27	7.53 ± 0.33
Rehabilitation department	8.06 ± 0.59	7.63 ± 0.41	7.29 ± 0.16	7.61 ± 0.25	7.51 ± 0.37

**Table 3:** The difference between the original AI/LMM-modified score and the expert score.

		Original MAE ^a^	LMM-modified MAE ^b^	*p*-Value
Overall				
	BERT	0.84 ± 0.27	0.70 ± 0.33	<0.001 *
	LSTM	1.00 ± 0.32	0.66 ± 0.39	<0.001 *
Internal medicine	BERT	0.82 ± 0.27	0.66 ± 0.37	0.007 *
	LSTM	0.96 ± 0.32	0.63 ± 0.41	<0.001 *
Surgery	BERT	0.86 ± 0.24	0.72 ± 0.25	0.011 *
	LSTM	1.04 ± 0.25	0.67 ± 0.30	<0.001 *
Obstetrics and pediatrics	BERT	1.05 ± 0.30	0.82 ± 0.32	0.069
	LSTM	1.21 ± 0.31	0.74 ± 0.44	<0.001 *
Other departments	BERT	0.79 ± 0.26	0.70 ± 0.35	0.142
	LSTM	0.96 ± 0.35	0.67 ± 0.41	<0.001 *
**Department**				
General surgery	BERT	0.80 ± 0.21	0.75 ± 0.15	0.645
	LSTM	1.03 ± 0.20	0.72 ± 0.12	0.003 *
Pleural surgery	BERT	0.72 ± 0.10	0.49 ± 0.26	0.200
	LSTM	0.91 ± 0.20	0.38 ± 0.27	0.100
Cardiovascular surgery	BERT	0.88 ± 0.26	0.86 ± 0.42	0.589
	LSTM	1.09 ± 0.39	0.79 ± 0.51	0.065
Colorectal & rectal surgery	BERT	0.74 ± 0.12	0.61 ± 0.25	0.686
	LSTM	0.97 ± 0.10	0.57 ± 0.34	0.057
Urology surgery	BERT	0.73 ± 0.06	0.67 ± 0.10	0.318
	LSTM	0.93 ± 0.10	0.63 ± 0.20	0.002 *
Plastic surgery	BERT	0.76 ± 0.05	0.59 ± 0.15	0.057
	LSTM	0.97 ± 0.08	0.52 ± 0.22	0.029 *
Pulmonary Medicine	BERT	0.94 ± 0.32	0.69 ± 0.29	0.040 *
	LSTM	1.14 ± 0.36	0.65 ± 0.27	0.002 *
Cardiology	BERT	1.01 ± 0.41	0.75 ± 0.33	0.136
	LSTM	1.12 ± 0.34	0.74 ± 0.34	0.024 *
Nephrology	BERT	0.89 ± 0.29	0.89 ± 0.41	0.841
	LSTM	1.22 ± 0.47	0.82 ± 0.49	0.222
Blood Oncology	BERT	0.85 ± 0.21	0.66 ± 0.23	0.130
	LSTM	0.91 ± 0.22	0.72 ± 0.28	0.195
Endocrine and metabolic	BERT	0.82 ± 0.03	0.68 ± 0.16	0.343
	LSTM	0.95 ± 0.09	0.63 ± 0.23	0.114
Gastroenterology	BERT	0.60 ± 0.11	0.42 ± 0.20	0.050 *
	LSTM	0.66 ± 0.17	0.37 ± 0.23	0.015 *
Rheumatism, immunology and allergy	BERT	0.74 ± 0.11	0.69 ± 0.13	0.548
	LSTM	1.02 ± 0.15	0.70 ± 0.16	0.032 *
Trauma	BERT	1.08 ± 0.22	0.88 ± 0.70	1.000
	LSTM	1.19 ± 0.63	0.84 ± 0.72	0.667
Infection and Tropical Medicine	BERT	0.69 ± 0.17	0.66 ± 0.81	0.028 *
	LSTM	0.78 ± 0.26	0.63 ± 0.91	0.028 *
Psychiatric department	BERT	0.73 ± 0.26	0.59 ± 0.47	0.328
	LSTM	1.03 ± 0.29	0.52 ± 0.54	0.028 *
Neurological department	BERT	0.72 ± 0.06	0.56 ± 0.06	0.002 *
	LSTM	0.82 ± 0.09	0.44 ± 0.11	0.002 *
Pediatric department	BERT	1.18 ± 0.35	0.95 ± 0.33	0.328
	LSTM	1.36 ± 0.30	0.90 ± 0.49	0.007 *
Dental department	BERT	0.96 ± 0.10	1.12 ± 0.24	0.400
	LSTM	1.52 ± 0.19	1.23 ± 0.23	0.400
ENT department	BERT	0.73 ± 0.13	0.53 ± 0.17	0.024 *
	LSTM	0.78 ± 0.15	0.46 ± 0.20	<0.001 *
Family and Community Medicine	BERT	0.75 ± 0.06	0.74 ± 0.43	0.700
	LSTM	0.80 ± 0.05	0.81 ± 0.62	0.700
Neurosurgery	BERT	1.12 ± 0.28	0.80 ± 0.10	0.002 *
	LSTM	1.21 ± 0.30	0.77 ± 0.14	0.002 *
Orthopedic department	BERT	0.78 ± 0.34	0.71 ± 0.38	0.630
	LSTM	0.92 ± 0.28	0.68 ± 0.42	0.089
Obstetrics and Gynecology	BERT	0.88 ± 0.03	0.64 ± 0.24	0.009 *
	LSTM	1.02 ± 0.19	0.53 ± 0.28	0.004 *
Ophthalmology department	BERT	0.56 ± 0.17	0.55 ± 0.26	0.690
	LSTM	0.60 ± 0.09	0.57 ± 0.30	0.222
Rehabilitation department	BERT	0.88 ± 0.12	0.77 ± 0.22	0.180
	LSTM	1.06 ± 0.38	0.77 ± 0.38	0.180

^a^ Original MAE: Expert’s score—BERT/LSTM prediction score. ^b^ LMM-modified MAE: Expert’s score—LMM-modified BERT/LSTM prediction score. *: *p*-value < 0.05.

**Table 4:** Experts’ scores, BERT and LSTM prediction scores, and MAE of different blocks.

Block	Experts’ Score (a)	BERT Score (b)	LSTM Score (c)	*p*-Value	LMM-Modified BERT Score (d)	LMM-Modified LSTM Score (e)	*p*-Value	|a-b| ^#^	|a-d| ^#^	*p*-Value	|a-c| ^#^	|a-e| ^#^	*p*-Value
A	7.44 ± 0.66	7.35 ± 0.17	6.99 ± 0.17	<0.001 *	7.08 ± 0.56	7.02 ± 0.66	0.626	0.83 ± 0.27	0.66 ± 0.38	0.008 *	0.97 ± 0.33	0.63 ± 0.43	<0.001 *
B	7.35 ± 0.51	7.43 ± 0.06	7.32 ± 0.17	0.087	7.32 ± 0.47	7.38 ± 0.54	0.824	0.7 ± 0.13	0.51 ± 0.17	0.013 *	0.76 ± 0.16	0.45 ± 0.2	0.002 *
C	7.88 ± 0.14	7.56 ± 0.09	7.4 ± 0.1	0.016 *	7.59 ± 0.18	7.63 ± 0.24	0.740	0.69 ± 0.03	0.54 ± 0.1	0.005 *	0.77 ± 0.08	0.41 ± 0.14	<0.001 *
D	7.94 ± 1	7.43 ± 0.19	7.13 ± 0.08	0.005 *	7.57 ± 0.6	7.61 ± 0.84	0.932	1.29 ± 0.29	0.88 ± 0.31	0.042 *	1.44 ± 0.3	0.74 ± 0.35	0.004 *
E	7.74 ± 0.91	7.51 ± 0.08	6.98 ± 0.18	<0.001 *	7.19 ± 0.45	7.12 ± 0.56	0.772	1.05 ± 0.33	0.85 ± 0.4	0.227	1.25 ± 0.41	0.8 ± 0.35	0.016 *
F	7.3 ± 0.63	6.97 ± 0.24	6.61 ± 0.17	0.004 *	6.75 ± 0.54	6.69 ± 0.74	0.874	0.88 ± 0.22	0.73 ± 0.28	0.258	1 ± 0.27	0.78 ± 0.3	0.154
G	7.76 ± 0.15	7.46 ± 0.08	7.08 ± 0.11	<0.001 *	7.52 ± 0.25	7.46 ± 0.37	0.707	0.69 ± 0.08	0.63 ± 0.12	0.238	0.93 ± 0.12	0.6 ± 0.2	0.003 *
H	8.41 ± 0.47	8.1 ± 0.06	8.05 ± 0.18	0.436	7.93 ± 0.59	7.95 ± 0.67	0.962	0.71 ± 0.23	0.58 ± 0.48	0.489	1 ± 0.3	0.51 ± 0.55	0.045 *

^#^: The mean absolute error (MAE), the absolute value of the original score minus the predicted score. *: *p*-value < 0.05.

## Data Availability

The data presented in this study are available on request from the corresponding author.
